# Global prevalence and predictors of depression and anxiety in patients with liver cirrhosis: a systematic review and meta-analysis

**DOI:** 10.3389/fepid.2026.1740227

**Published:** 2026-04-17

**Authors:** Omar Abureesh, Araek Al-Shraideh, Joelle Sleiman, Chloe Lahoud, Brendan Plann-Curley, Liliane Deeb

**Affiliations:** Northwell Health, Staten Island, Staten Island University Hospital, Staten Island, NY, United States

**Keywords:** alcoholic liver, anxiety, cirrhosis, depression, non-alcoholic fatty liver disease, viral hepatitis

## Abstract

**Background:**

Liver cirrhosis is a complex disorder that affects nearly 122 million patients worldwide. This study synthesizes global prevalence estimates of depression and anxiety among patients with cirrhosis, together with associated risk factors and geographic distribution.

**Method:**

An electronic search was conducted on Medline, Embase, Cochrane Central and Web of Science databases. Results were then filtered according to the inclusion criteria over two stagesData from eligible studies were extracted into a standardized spreadsheet, which was then subjected to analysis and evidence synthesis.

**Results:**

Our search yielded 23 articles from countries all over the world describing 979,113 patients.The pooled prevalence was 0.37 [95% C.I. 0.29–0.46, *p* = 0.01] for depression and of 0.53 [95% C.I. 0.33–0.73, *p* < 0.010] for anxiety, in cirrhotic patients, however, high heterogeneity was noted. Meta-regression was performed to assess the ability of demographic factors (Ager, Sex), and etiological factors to predict depression in cirrhotic patients. Age, alcoholism, and viral etiologies, were linked to depression incidence. Advancing age was associated with increased depression prevalence among cirrhosis patients (*p* = 0.02).

**Conclusion:**

Depression and anxiety substantially impair quality of life in patients with cirrhosis, but their diagnosis remains limited and under-investigated. Standardizing depression and anxiety screening for cirrhosis patients can improve their outcomes and quality of life significantly.

## 1 Introduction

Liver cirrhosis results from chronic hepatic inflammation and injury and accounts for approximately 2.4% of global deaths annually. According to the Global Burden of Diseases, Injuries, and Risk Factors Study (GBD), cirrhosis affects nearly 122 million patients worldwide (1). Cirrhosis starts with liver inflammation that is followed by diffuse hepatic fibrosis, wherein the normal hepatic architecture is replaced by regenerative hepatic nodules, which eventually leads to liver failure. Disease course varies from weeks to decades (2). Common causes of cirrhosis include chronic hepatitis B and C, alcohol-related liver disease, and non-alcoholic steatohepatitis (NASH) (3). Liver cirrhosis typically begins in a compensated stage, during which most patients remain asymptomatic. Over time, depending on the underlying etiology, the disease may progress to decompensation, defined by the first occurrence of complications associated with portal hypertension or impaired hepatic function, such as ascites, hepatic encephalopathy, or gastrointestinal bleeding. At this point, cirrhosis is clinically categorized into two stages: a compensated stage, usually associated with preserved quality of life, and a decompensated stage, characterized by major complications and a reduced median survival of approximately 2–4 years (4, 5). Beyond physical complications, cirrhosis imposes significant psychological burden driven by chronic symptoms, social stigma, financial stress, and neurocognitive dysfunction, contributing substantially to depression and anxiety and reducing treatment adherence (6). Depression and anxiety compromise patients’ mental health, reduce treatment adherence and contributes to a vicious cycle of worsening psychiatric symptoms and disease-related complications.

Numerous studies have reported associations between cirrhosis and depression or anxiety (7–9); however, prevalence estimates vary widely. Moreover, the factors that influence this prevalence remain of need for analysis and recognition. This systematic review aims to collect and analyze the global prevalence of depression and anxiety among cirrhotic patients along with the associated risk factors and geographical distribution.

## 2 Methods

### 2.1 Study design

This study was conducted in accordance with the Preferred Reporting Items for Systematic Review and Meta-analysis (PRISMA), and Northwell Health Libraries (10). A pre-defined protocol was designed and validated by senior authors.

### 2.2 Search strategy

The search was conducted by a health sciences librarian, and results were uploaded to Covidence for review by authors. Comprehensive searches were performed on Medline, Embase, Cochrane Central and Web of Science databases on July 15, 2025, from database inception onward. The following keywords were used to identify relevant articles: (“cirrhosis of the liver” OR “chronic liver diseases” OR “cirrhosis” OR “Liver Fibrosis”) AND (“depression” OR “Anxiety” OR “Major depressive disorder” OR “MDD” OR “Melancholia” OR “Depressive Disorder” OR “Anxiety” OR “Anxiety Disorders” OR “Generalized Anxiety Disorder”). Additionally, all potentially pertinent papers were found using Medical Subject Headings (Mesh) terms based on these indexed terms. After the articles were screened, we manually searched for any potentially missing pertinent articles using three methods to ensure a comprehensive search: (a) screening the included articles’ reference list; (b) screening “similar articles” to the included ones using PubMed’s “similar articles” options; and (c) examining the included articles in the earlier meta-analysis.

### 2.3 Eligibility criteria

Studies that satisfied the following requirements were included: [1] Adults (>18 years) with liver cirrhosis [2] Patients with anxiety and/or depression [3] English papers were recruited. There were no limitations on the publication date. However, studies that met any of the following criteria were not included. [1] Individuals without a cirrhosis or chronic liver disease diagnosis, [2] Individuals without a depression or anxiety diagnosis [3] Individuals not concurrently diagnosed with cirrhosis and anxiety or depression.[4] Children (less than 18 years old) [5] publications written in languages other than English [6], models that are not human, and [7] entire texts that are not accessible.

### 2.4 Study selection

Citations were entered into EndNote to eliminate duplicates once the studies were retrieved from the database search, and then they were exported into an Excel sheet for screening. Initially, retrieved study titles and abstracts were compared to our predetermined eligibility standards. Next, full-text screening was applied to possibly pertinent studies. Two sets of two independent reviewers [O.A., A.A.] completed this process, and any disagreements were resolved by consulting a third reviewer [S.J.]. Cohort studies, case control studies, and randomized control trials (RCTs) were among the original articles that were taken into consideration for inclusion; case reports, case series, letters to editor, secondary research as systematic reviews, literature reviews and meta-analyses, and commentaries were not. The frequency of anxiety or depression in individuals with liver cirrhosis should be reported in the included studies.

### 2.5 Data extraction

The primary outcome of interest was the prevalence of depression, and/or anxiety in patients with liver cirrhosis. Study characteristics, patient characteristics, proportion of patients with liver cirrhosis, methods of diagnosis of the liver cirrhosis, severity upon diagnosis, proportion of patients with depression and/or anxiety and management of each were extracted into a common table. To handle differences in diagnostic methods, data on utilized scales or tools was collected, along with the values measured.

### 2.6 Quality assessment

The methodological quality of the included studies was evaluated using the Newcastle Ottawa scale for risk of bias assessment and the high risk of bias is considered at score 60% score. This process was carried out by two sets of two reviewers [O.A., A.A. and J.S., L.D.], and discrepancies were solved by group discussion.

### 2.7 Data synthesis

The collected data were analyzed by quantitative and qualitative methods. Patient and study characteristics were reported narratively in tables. Continuous data were reported as mean and standard deviation, and nominal data were reported as counts and percentages. A fixed-effect model meta-analysis with proportion as the effect measure and 95% confidence intervals (CI) were used to examine the proportions of anxiety and depression. Scores were analyzed using standardized mean difference to account for scale variability the measurement of effect. *I*^2^ and Cochran’s Q statistic were used to measure heterogeneity, and they were interpreted in accordance with the Cochrane Handbook for Systematic Reviews (8). We used a random-effects model (DerSimonian and Laird) when significant heterogeneity (*I*^2^ > 50%) was found. The threshold for statistical significance was established at *p* < 0.050. We used the leave-one-out technique to find research that caused heterogeneity. The relevant study was removed from the synthesis if considerable heterogeneity remained after converting to a random-effects model. R software (version 4.12-0 of the meta package; R Foundation for Statistical Computing; Vienna, Austria) was used to perform statistical analyses. Meta-analysis of proportions could not be used to analyze publication bias using funnel plot asymmetry.

## 3 Results

### 3.1 Search results and included studies

**Figure 1:**
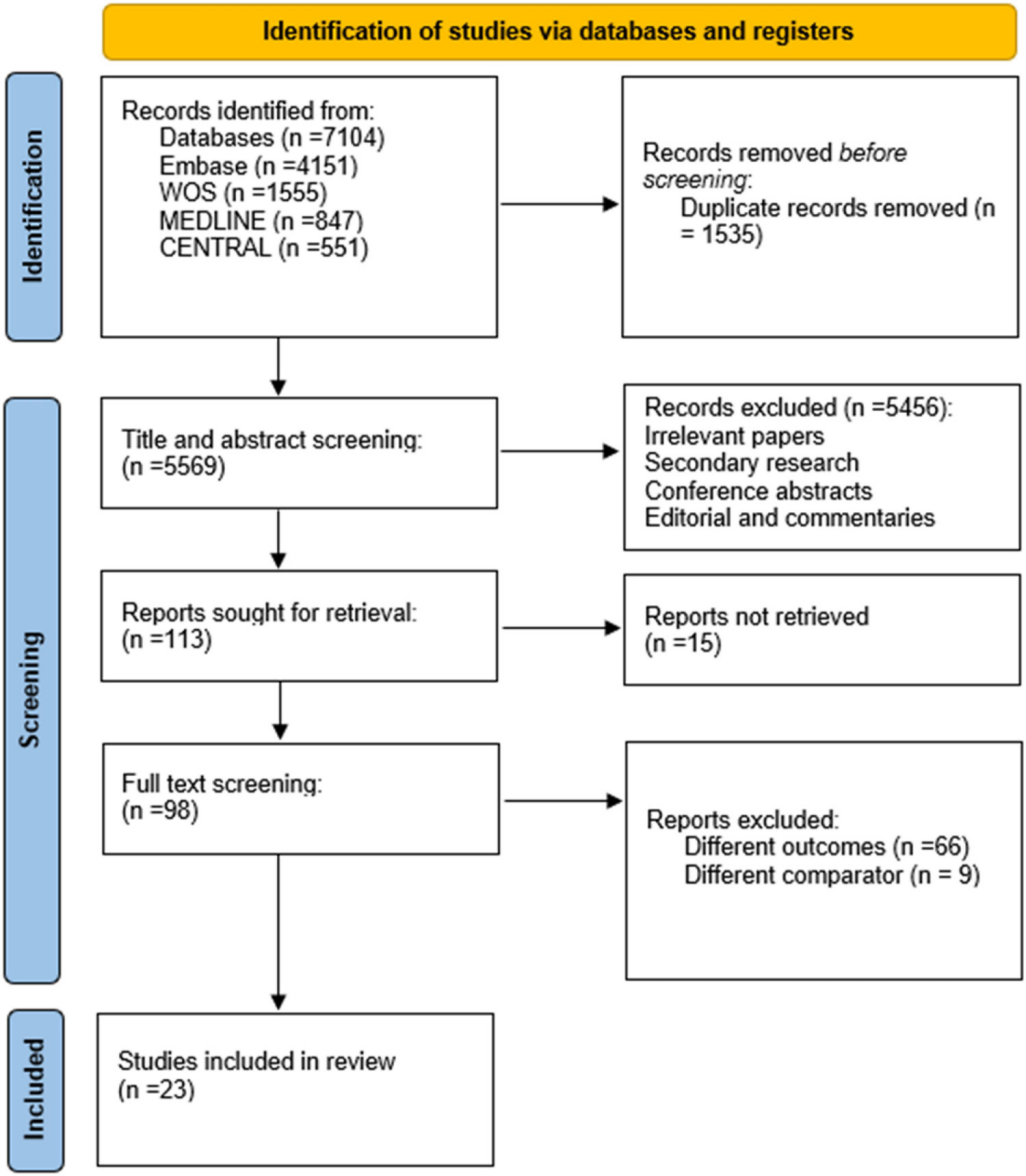
Prisma flow diagram of the filtration process.

Of the 7,104 articles identified by the search, 1,535 were removed as duplicates, 5,265 were excluded during title and abstract screening due to failure to meet inclusion criteria, and 272 more were excluded upon full text review for failure to meet inclusion criteria. Our search yielded 23 articles from all over the world describing 979,113 patients. Included articles investigated the relationship between cirrhosis and depression or anxiety in 978,159 (99.9%) patients. Details of the screening process are presented in Figure 1.

### 3.2 Study and population characteristics

Eleven studies were cohort designs, either retrospective or prospective, while the remaining 12 studies were of cross-sectional methodology. Most studies included were hospital-based studies (82.6%), on the other hand; 4 studies were population-based large cross-sectional studies. The mean age of participants was 57.1 ± 13.4 years, and all genders were represented almost equally (Male% = 57%). Table 1 describes the characteristics of included studies and the demographics of their populations.

**Table 1:** Characteristics of included studies.

ID	Country	Method	Cohort type	Sample Size	Cirrhosis patients (%)	Age	Sex (M%)
Østberg et al. (11)	Denmark	Retrospective Cross sectional	Hospital cohort	340	74 (21.76%)	60.7 ± 12.6*	204 (60%)
Seo et al. (12)	South Korea	Retrospective Cohort	Population based	510,737	510,737 (100%)	55.8 ± 13.5	349,344.108 (68.4%)
Abureesh et al. (13)	USA	Retrospective Cohort	Population based	293,150	293,150 (100%)	Not reported	Not reported
Hernaez et al. (8)	USA	Cross sectional	Hospital cohort	1,021	1,021 (100%)	58.3 ± 11.5*	652.419 (63.9%)
Buganza-Torio et al. (14)	Canada	Prospective Cohort	Hospital cohort	305	305 (100%)	55 ± 10	198.25 (65%)
Zhang et al. (15)	China	Cross sectional	Hospital cohort	1,736	1736 (100%)	53 ± 12.0*	937.44 (54%)
Perng et al. (16)	Taiwan	Retrospective Cohort	Population based	52,725	52,725 (100%)	58.7 ± 17.0*	33,005.85 (62.6%)
Kalaitzakis et al. (17)	Sweden	Prospective Cohort	Hospital cohort	108	108 (100%)	53 ± 8	75.6 (70%)
Aghanwa et al. (18)	Nigeria	Prospective Cohort	Hospital cohort	31	31 (100%)	44.3 ± 13.1	19.003 (61.3%)
Bona et al. (19)	Italy	Prospective Cross sectional	Hospital cohort	141	141 (100%)	47 ± 7.5*	98.7 (70%)
Singh et al. (20)	USA	Prospective Cohort	Hospital cohort	81	81 (100%)	46 ± 7.7*	80.19 (99%)
Malik et al. (21)	Pakistan	Cross sectional	Hospital cohort	382	382 (100%)	Not reported	223.852 (58.6%)
McPhail et al. (22)	Australia	Prospective Cohort	Hospital cohort	562	562 (100%)	59.8 ± 11	392.838 (69.9%)
Seo et al. (23)	South Korea	Cross sectional	Hospital cohort	80	80 (100%)	Not reported	61.04 (76.3%)
Barboza et al. (24)	USA	Cross sectional	Hospital cohort	43	43 (100%)	57.8 ± 7	27.09 (63%)
Benzing et al. (25)	Germany	Cross sectional	Hospital cohort	292	195 (66.78%)	56.0 ± 9.0	18,512.8 (63.4%)
Jafree et al. (26)	Pakistan	Cross sectional	Hospital cohort	850	259 (30.47%)	Not reported	42,840 (50.4%)
Donlan et al. (27)	USA	Retrospective Cohort	Population based	115,409	115409 (100%)	61.9 ± 9.0*	111,369.685 (96.5%)
Bianchi et al. (28)	Italy	Cross sectional	Hospital cohort	156	156 (100%)	65 ± 8.3*	87.36 (56%)
Ufere et al. et al. (29)	USA	Cross sectional	Hospital cohort	127	127 (100%)	59 ± 9.6*	69.85 (55%)
López-Navas et al. (30)	Spain	Cross sectional	Hospital cohort	63	63 (100%)	Not reported	Not reported
Dang et al. (31)	Canada	Prospective Cohort	Hospital cohort	304	304 (100%)	56 ± 10	223.44 (73.5%)
Liang et al. (32)	China	Retrospective Cohort	Hospital cohort	470	470 (100%)	58.7 ± 12	427.7 (91%)
Total				979,113	978,159 (99.9%)	57.1 ± 13.4	558,851.22 (57%)

* SD derived from IQR or range via Cochrane-recommended conversions.

### 3.3 Clinical features

Patients exhibited heterogeneous cirrhosis etiologies for the development of cirrhosis. Most patients were diagnosed with cirrhosis without a clear etiological factor (85.2%). Among common causes of liver cirrhosis, non-alcoholic or cryptogenic liver disease had the highest prevalence (8.25%), which was significantly correlated to the diagnosis of depression (*p* < 0.010), followed by alcoholic liver disease (3.98%), which showed a borderline non-significant association which showed a borderline non-significant association (*p* = 0.06). Viral hepatitis was reported in (2.55%) of patients and was not significantly associated with either diagnosis. Other causes were less frequently reported. Diagnosis of liver cirrhosis can be made by utilizing several tools. In included articles, 21 (91.3%) articles used clinical assessment for diagnosis. Imaging techniques were less utilized, used in 10 (43.48%) of included studies, followed by biopsy and laboratory investigations, both used in 7 (30.43%) of studies. Tables 2, 3 describe the clinical and diagnostic information of included studies.

**Table 2:** Clinical etiology of cirrhosis in included patients.

Etiology	*N* (%)	*P*-value xDepression	*P*-value xAnxiety
Alcoholic Hepatitis	38,963 (3.98%)	0.06	0.25
NASH/Cryptogenic	80,653 (8.25%)	<0.010	0.78
Viral Hepatitis (HBV, HCV, HDV)	24,970 (2.55%)	0.68	0.70
Autoimmune	123 (0.01%)	0.80	0.67
Cholestasis	40 (0%)	–	<0.010
HCC	14 (0%)	–	–
Other/ Unspecified	833,396 (85.2%)	<0.010	0.10

**Table 3:** Cirrhosis diagnosis and severity assessment.

Diagnosis	*N* (%)
Modality	
Clinical	21 (91.3%)
Imaging	10 (43.48%)
Biopsy	7 (30.43%)
Labs	7 (30.43%)
Severity assessment	
Both	3 (12.5%)
Child-Pugh score	10 (41.7%)
Child-Pugh (A)	1,421 (0.15%)
Child-Pugh (B/C)	1,591 (0.16%)
CLDQ subscales	1 (4.2%)
MELD score	2 (8.3%)
Mean ± SD	9.53 ± 1.79
Total	24 (100%)

### 3.4 Depression

**Figure 2:**
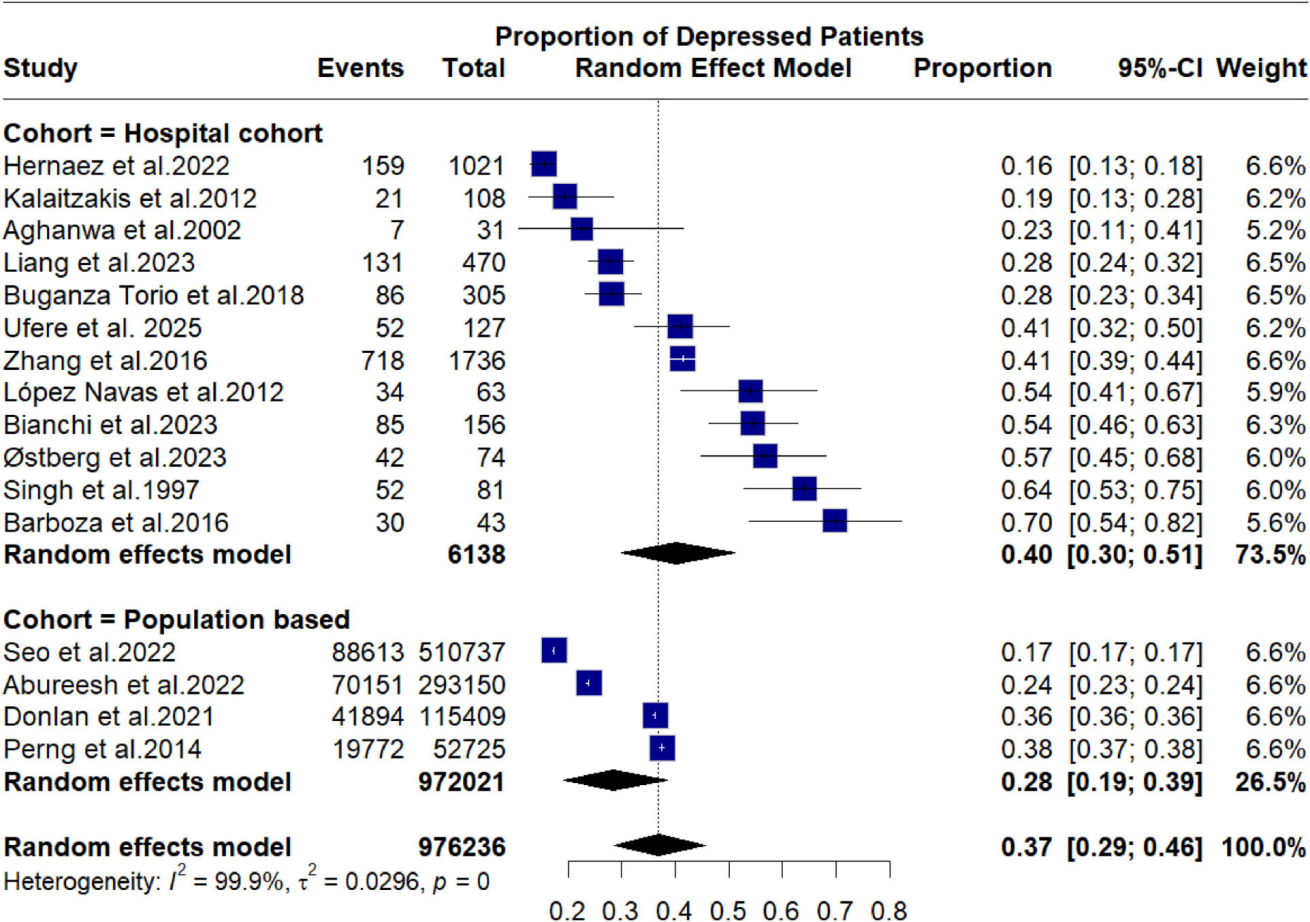
Forest plot showing random effect mode analysis of depression proportions.

A meta-analysis of proportions was conducted to assess the proportion of depression and anxiety in cirrhotic patients. The subgroup analysis shown in Figure 1 illustrates an overall proportion of depression of 0.37 [95% C.I. 0.29–0.46, *p* = 0.01] in cirrhotic patients. In hospital-based studies, the proportion of depression was 0.4 [95% C.I. 0.3–0.51], while in population-based studies, the proportion decreased to 0.28 [95% C.I. 0.19–0.39]. Results show high rate of heterogeneity, (*I*^2^ = 99.9%), and sensitivity analysis failed to identify a particular source for heterogeneity, and was therefore attributed to inherent variability across source studies reflecting original source data variability. Meta-regression was performed to assess the ability of demographic factors (Ager, Sex), and etiological factors to predict depression in cirrhotic patients. Age, alcoholic, and viral etiologies significantly predicted depression incidence, although the estimate values were small. Figure 2 shows the forest plot of depression proportion, while Table 4 illustrates meta-regression of depression factors.

**Table 4:** Meta-regression of depression factors.

Factor	Estimate	SE	Zval	*P*-value	95% C.I.
Intercept	−0.55	0.64	−0.86	0.40	[−1.8:0.71]
Age	0.02	0.01	2.25	0.020	[0.003:0.04]
Sex	0.01	0.47	0.01	0.99	[−0.92:0.93]
Alcoholic-liver	0.0005	0.0002	2.647	<0.010	[0.0001:0.0008]
NASH	0	0	−1.2203	0.22	0
Viral	−0.0007	0.0003	−2.6265	<0.010	[−0.0013: −0.0002]
AIH	−0.01	0.0031	−1.72	0.090	[−0.01:0.0007]
Biliary	−0.004	0.01	−0.42	0.67	[−0.02:0.01]
HCC	0.03	0.02	1.49	0.14	[−0.01:0.06]
Other	0	0	−2.3931	0.020	0

### 3.5 Anxiety

**Figure 3:**
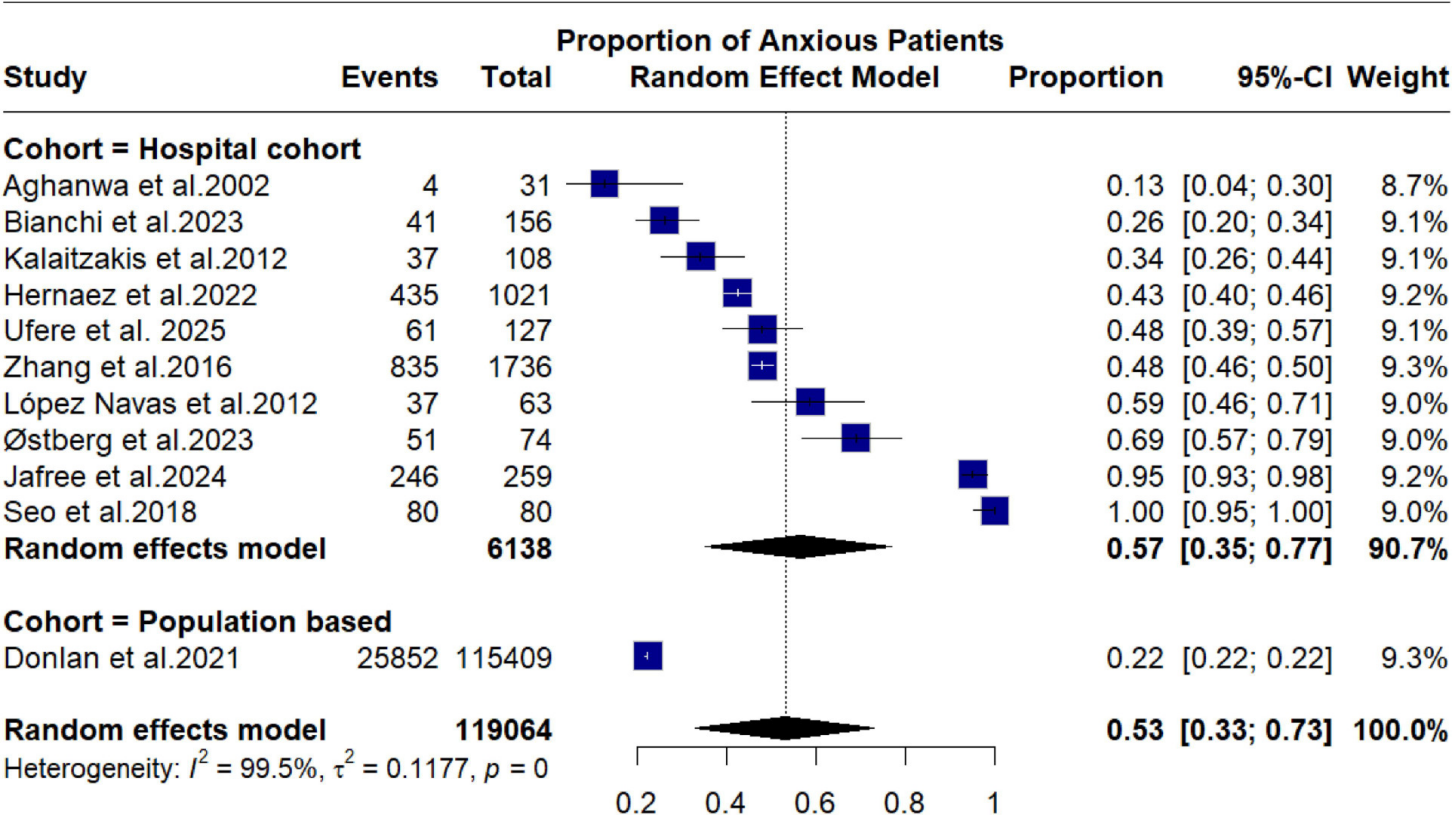
Forest plot showing random effect mode analysis of anxiety proportions.

Anxiety proportions were assessed using meta-analysis of proportions. Only one population-based study reported anxiety incidences in its cohort. Among studies reporting anxiety incidences, the overall proportion was 0.53 [95% C.I. 0.33–0.73], with significant subgroup differences (*p* < 0.010). This proportion was mainly due to hospital-based cohorts anxiety incidence was 0.57 [95% C.I. 0.35–0.77]. Significant heterogeneity was observed as well, with *I*^2^ = 98.5%. Like depression estimates, data showed a lot of variability, which reflects heterogeneity in primary datasets. Meta-regression of factors suspected to predict incidence of anxiety was conducted without significant results. Figure 3 shows the forest plot of anxiety proportion, while Table 5 illustrates meta-regression of anxiety factors.

**Table 5:** Meta-regression of anxiety factors.

Factor	Estimate	SE	Zval	*P*-value	95% C.I.
Intercept	0.46	0.64	0.73	0.47	[−0.7851:1.7139]
Age	0.01	0.01	0.98	0.33	[−0.0106:0.0317]
Sex	−0.63	0.47	−1.32	0.19	[−1.556:0.3033]
Intercept	0.92	0.27	3.37	0.0008	[0.38:1.45]
Alcohol	0.0041	0.01	0.56	0.58	[−0.01:0.02]
NASH	−0.01	0.01	−0.51	0.61	[−0.03:0.02]
Viral	−0.0003	0.001	−0.3	0.77	[−0.0023:0.0017]
AIH	−0.02	0.02	−0.98	0.33	[−0.06:0.02]
Biliary	−0.02	0.03	−0.87	0.38	[−0.07:0.03]
Other	−0.0001	0.0003	−0.18	0.86	[−0.0006:0.0005]

### 3.6 Risk of bias assessment

**Figure 4:**
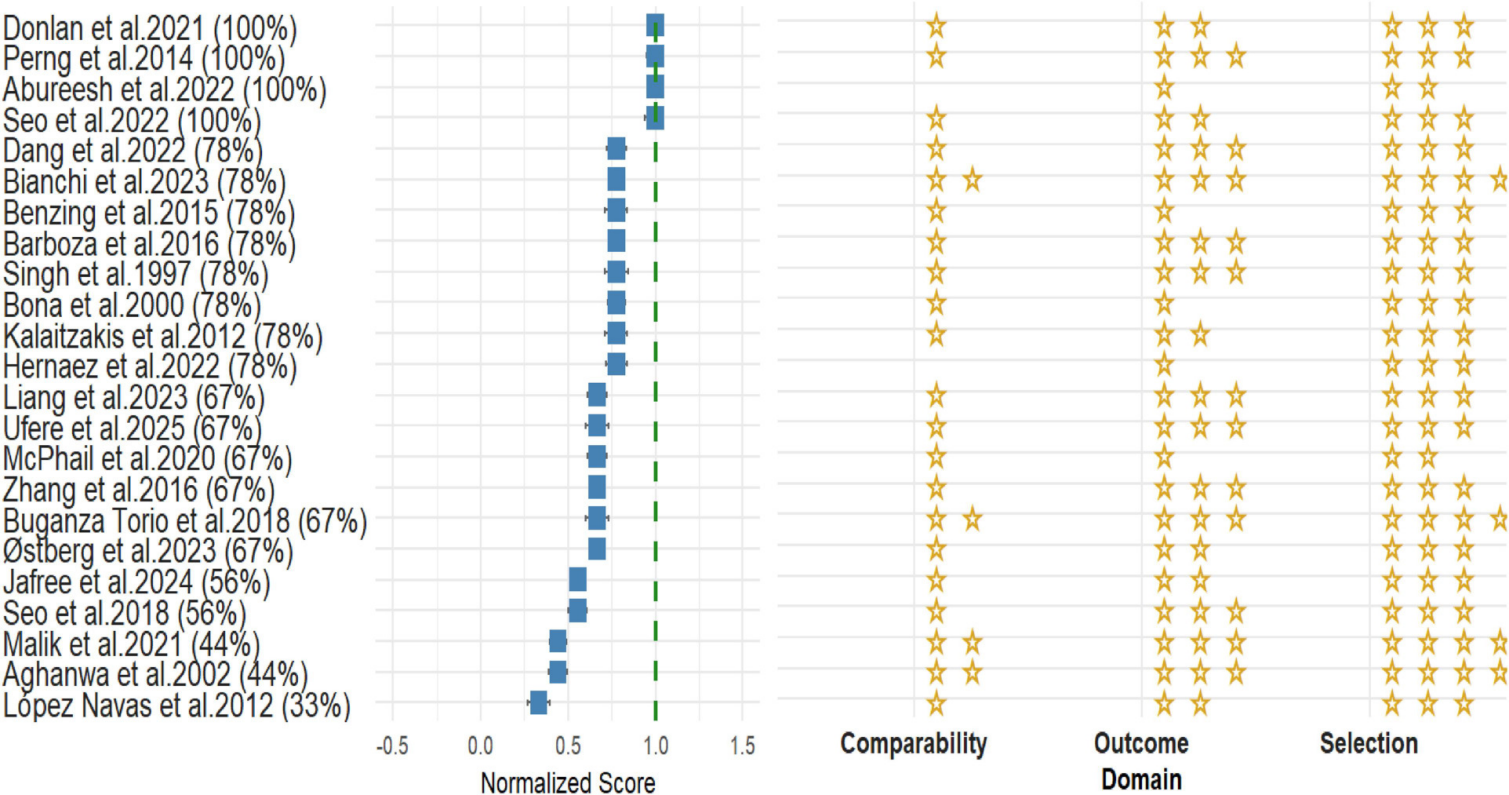
Newcastle-Ottawa risk of bias assessment of included studies.

The Newcastle Ottawa scale for risk of bias assessment was utilized to assess included studies. Five of the studies included were suspected of high risk of bias (<60% score), the remaining articles were found of moderate to low risk of bias. Figure 4 illustrates the individual risk of bias assessment for each of the included articles. Analysis was reattempted after excluding high risk studies, and results remained largely consistent across outcomes. For depression, the outcome was 0.36 [95% C. I.: 0.28; 0.45. *I*^2^ = 99.9%], and for anxiety, we found 0.38 [95% C.I.: 0.26; 0.5, *I*^2^ = 99.1%].

## 4 Discussion

Depression and anxiety are more prevalent in cirrhosis patients than they are in the general population and increasing rates of depression and anxiety among cirrhotic patients have been suggested in recent years (9). The present study provides insight into the prevalence of these psychiatric issues in patients with liver cirrhosis, since the chronic disease has been widely linked to both depression and anxiety (33).

This review synthesized data from nearly one million patients. Of those, 978,159 (99.9%) patients were investigated for depression and anxiety after the diagnosis of cirrhosis was established. Cirrhosis in these patients was due to various factors including alcohol, nonalcoholic fatty liver disease (NAFLD) and viral hepatitis, however, the majority of records did not specify the underlying etiology (85.2%). Among identified cirrhosis causes, significant correlation was observed between depression and NAFLD (8.25%, *p* < 0.010), while anxiety was associated with cholestasis (*n* = 40, ≈0%, *p* < 0.050). This finding contrasts with global data indicating viral hepatitis B and C as the most prevalent causes of cirrhosis, with a global prevalence of 42% and 21% respectively (34). The majority of included patients in the present study are either in the USA or South Korea, both of which have a higher proportion of NAFLD-induced liver cirrhosis. This can explain the discrepancy between global etiological estimates and the results presented in this study since the USA alone has NAFLD prevalence of 20% among all cirrhosis cases (35). Moreover, since this review focuses on depression and anxiety, and both conditions have higher prevalence in the US population compared to global rates, the geographic focus may have influenced the demographic distribution of patients included in this analysis (36–39). Similar to our findings, a significant correlation between NAFLD and depression has been reported in the literature. A recent meta-analysis by Gu et al. found that NAFLD patients are at an increased risk of developing clinical depression with odds of 1.13 (*p* < 0.010) (40).

The overall pooled estimate of depression among cirrhosis patients included in our study was found at 37% [95% C.I. 0.29–0.46, *p* = 0.01], with significantly heterogeneous data. This heterogeneity can be attributed to the clinical nature of cirrhosis and psychiatric disorders. The extreme heterogeneity limits the accuracy and generalizability of pooled prevalence estimates and calls for cautious interpretation of summary effects. This is probably due to significant variation across studies in cirrhosis diagnostic criteria, psychiatric screening tools, disease stage, healthcare access, and reporting of etiological factors. Therefore, this estimate provides an approximal global burden rather than epidemiological prevalence. On one hand, cirrhosis is a complex issue that is affected by several factors including general health conditions, nutrition, genetics, and behavior. On the other hand, depression and anxiety are also complex disorders that are often impacted by individual, cultural, and behavioral patterns. These complexities, in addition to varying levels of healthcare access and assessment availability, would naturally result in data with limited consistency across the world (9). Factors that have been investigated using meta-regression include age, gender, and etiology. Statistical significance was observed positively with age and alcoholic liver disease, while significant negative association was noted with viral etiology. The only clear factor is the age of patients, and our estimates suggest that advancing in age with cirrhosis corresponds to a higher risk of developing depression. These findings were supported by a study on cirrhosis and depression and anxiety by Zimbrean and Jakab, which suggested that age > 65 is significantly associated with depressive symptoms (9). Etiological factors that were significantly associated with developing depression had very low estimates, which suggest that although they were statistically significant, they do not hold any clinical impact on the risk of depression in cirrhotic patients. In clinical settings, these variables may not be of considerable effect regarding depression development in cirrhosis patients. Once established, cirrhosis exerts substantial burden on patients regardless of etiology.

As for anxiety, the overall proportion was 53% [95% C.I. 0.33–0.73], with significant data heterogeneity as well. Similar to depression, this estimate is burdened by the heterogeneity in data, thus, it should be used as an estimate of global burden rather than epidemiological prevalence. As a result, hospital-based cohorts account for nearly all anxiety prevalence estimates, which significantly restricts population-level inference and highlights the limited body of data supporting anxiety outcomes in cirrhosis. Clinical diseases have been linked to anxiety disorders to a lower extent in literature. A study in anxiety levels in chronic disease patients found a pooled prevalence of 13.7% (33). However, the nature of liver cirrhosis can be terminal, subjecting patients to higher levels of fear and anxiety. The study by Zimbrean and Jakab found that the prevalence of anxiety among cirrhotic patients ranges from 10%–79% depending on the severity of the disease upon diagnosis (9). Another study on NAFLD and psychiatric issues concluded anxiety prevalence of 37.2%, and stress prevalence of 51.4% (41). These variations suggest that information available on this matter is inconclusive, and a clear-cut criteria for anxiety detection and reporting should be adopted to clarify the correlation between cirrhosis and anxiety disorders. Meta-regression was performed to investigate factors associated with increased or decreased anxiety occurrence. Unfortunately, none of the factors investigated resulted in a significant association.

The present review remains limited. The quality of included studies was generally acceptable, except for five studies which were found to have high risk of bias (18–23). This highlights the need for higher level evidence to be synthesized. Another limitation is the heterogeneity present in the included data. This matter can be attributed to the diversity of inclusion criteria. Extreme heterogeneity (*I*^2^ ≈ 99%), underreporting of cirrhosis etiology, mixed psychiatric assessment tools, and inclusion of high-risk studies substantially limit certainty. Cirrhosis diagnosis and depression and anxiety assessment should be standardized to avoid divergence, and to allow for cross-study analysis. Attempted to reduce heterogeneity by excluding high risk studies were unsuccessful, further emphasizing that the inherent variability in data due to clinical and diagnostic differences is the reason. Moreover, a need for controlled studies is present, since controlled studies can help better identify both causing and correlating factors for developing depression and anxiety in cirrhotic patients. In the present review, studies based on large registries reported likely stabilized prevalence estimates (13–16) (27). We recommend future investigations to adopt a clear diagnostic criterion for cirrhosis, based-on universally agreed upon staging criteria like MELD or Child-Pugh staging. Also, a unified tool for depression and anxiety assessment is needed. Scores like PHQ-9 and GAD-7 should be adopted. We also recommend conducting primary investigations on large cohorts to provide solid high-quality evidence. Prospective multicenter cohort studies integrating hepatology and psychiatry should be conducted, and future studies should focus on stratification by disease stage and etiology in future analyses. In addition, routine mental health screening embedded into cirrhosis clinics are necessary.

## 5 Conclusion

Depression and anxiety are associated with reduced quality of life in patients with liver cirrhosis. We found a pooled depression proportion of 0.37 [95% C.I. 0.29–0.46, *p* = 0.01] among cirrhosis patients, while pooled anxiety proportion of 53% [95% C.I. 0.33–0.73, *p* < 0.010] was observed for the same population, however, heterogeneity was substantial, limiting certainty in these findings. Advanced age was the only attributable factor that significantly increased depression prevalence in patients with cirrhosis, while none of the demographic or etiological factors was found to significantly affect the prevalence of anxiety. The issue of depression and anxiety in cirrhosis patients requires further high-quality investigation and research.

## Data Availability

The data analyzed in this study is subject to the following licenses/restrictions: dataset well be provided when requested from the author directly. Requests to access these datasets should be directed to oabureesh@northwell.edu.
